# Risk and Protective Factors of Generalized Anxiety Disorder of Elite Collegiate Athletes: A Cross-Sectional Study

**DOI:** 10.3389/fpubh.2021.607800

**Published:** 2021-02-10

**Authors:** Chunxiao Li, Rong Fan, Jian Sun, Guoxing Li

**Affiliations:** ^1^School of Physical Education and Sports Science, South China Normal University, Guangzhou, China; ^2^School of Physical Education and Sports, China University of Geosciences, Wuhan, China; ^3^School of Physical Education, Central China Normal University, Wuhan, China; ^4^Faculty of Athletic Training, Guangzhou Sport University, Guangzhou, China

**Keywords:** sport, mental disorder, predictor, university, observational study

## Abstract

The aim of this study was to examine the prevalence of Generalized Anxiety Disorder (GAD) and its risk and protective factors in elite collegiate athletes. A cross-sectional survey was conducted during the 2019 in-season. A sample of elite collegiate athletes (*n* = 285) from China completed a self-report form assessing GAD and potential predictors including age, gender, sport type, sport achievement, sport injury, attention deficit hyperactivity disorder (ADHD), fear of failure, mental toughness, and satisfaction in sport. The overall prevalence of GAD symptoms was 22%. The results of zero-order correlation showed that age, gender, sport type, and sport achievement were not significantly related to GAD. However, athletes with a history of sport injury, a high risk of ADHD, and a high level of fear of failure had a significant and positive association with GAD (*r* = 0.14–0.54). Meanwhile, high levels of mental toughness and satisfaction in sport were significantly and negatively related to GAD (*r* = −0.22 to −0.24). The results of multiple regression analysis indicated that sport injury, ADHD, and fear of failure were significant risk factors of GAD (β = 0.10–0.40). These findings suggest the necessity to understand the GAD symptoms in elite collegiate athletes. Further research is needed to better understand and support the mental health of this target group.

## Introduction

The sport community has paid increased attention to the mental health of elite athletes (1). Elite athletes refer to those who compete at the collegiate, Olympic, or professional levels (2). While sports participation can bring lots of benefits to elite athletes, they usually need to cope with both sport-specific and non-sport-specific demands (e.g., education, performance expectation, and sport injuries), which can pose potential threats to their mental health (3, 4). According to a recent meta-analysis (5), the prevalence of mental disorders and symptoms in elite athletes ranged from 19% for alcohol misuse to 34% for anxiety/depression.

Anxiety disorders refer to a group of disorders manifesting as excessive fear, tension, and worry (6). These disorders include different subtypes, such as agoraphobia, Generalized Anxiety Disorder (GAD), and social anxiety disorder. Anxiety disorders have been found to deteriorate sport performance (7), suggesting the need to understand their risk and protective factors.

Several factors may result in the development of anxiety disorders in elite athletes. For example, female and younger athletes tended to have a higher level of GAD than male and older athletes (8). Other potential risk factors for anxiety disorders in elite athletes include career dissatisfaction, sport injury, and adverse life events (2, 9, 10). However, little is known about other predicting factors of anxiety disorders, such as sport type, sport achievement, attention deficit hyperactivity disorder (ADHD), mental toughness (the ability to deal with adversity), and fear of failure (a tendency to avoid associated shame after one fails). For example, sport type, ADHD, and fear of failure have been identified as predictors of mental health problems other than anxiety disorders, such as burnout, depressive symptoms, and obsessive compulsive disorder (8, 11–13). Furthermore, only a few of these outlined factors have been examined within the same study. Thus, it is unclear which factor plays a more significant role in predicting anxiety disorders than the other.

The aim of this research was to examine the prevalence of GAD and its associated risk and protective factors (i.e., gender, age, sport type, sport achievement, sport injury, ADHD, mental toughness, fear of failure, and satisfaction in sport) in elite collegiate athletes. According to the above literature review, it was hypothesized that female, younger age (8), individual sport (11), ADHD (14), sport injury (2), and fear of failure (15) would be risk factors of GAD. In contrast, sport achievement (16), mental toughness (17), and satisfaction in sport (2) would be protective factors.

## Materials and Methods

### Design

A cross-sectional survey design was used in the present study. Ethics approval for the study was obtained from the Human Research Ethics Committee of China University of Geosciences (Wuhan) on 19 September 2019.

### Setting and Participants

Participants must meet the following inclusion criteria: (a) currently an active collegiate athlete, (b) based in a public university in China, (c) within the age range of 16–30 years, and (d) a grade 2 or above athlete (i.e., generally compete at provincial level or above). Athletes are classified into six levels based on their sport achievement under the Chinese sport system: international master sportsman, national master sportsman, grade 1, grade 2, grade 3, and no grade. Head coaches from four public universities located in Hubei and Zhejiang provinces were approached to invite eligible athletes to participate in this anonymous survey between September and December 2019 (i.e., in-season). A total of 337 athletes were invited, and 289 of them completed the paper-and-pencil-based survey (86%). Four athletes were excluded as they were grade 3 or non-grade athletes. Thus, the data of the remaining 285 athletes were used for analysis.

### Measures

The outcome of the present research was GAD. Risk and protective factors included demographic variables (i.e., age, gender, sport type, and sport achievement), sport injury, ADHD, fear of failure, mental toughness, and satisfaction in sport.

#### GAD

The validated Chinese version of the GAD-7 scale was used to measure GAD symptoms (18). The 7-item scale was developed based on the criteria stated in the fifth version of the Diagnostic and Statistical Manual of Mental Disorders (DSM-5). A 4-point scale (0 = not at all, 3 = nearly every day) was used for rating the items (e.g., “Over the last 2 weeks, how often have you been bothered by feeling nervous, anxious, or on edge”). A total score ≥10 out of 21 was used as a diagnostic cutoff for GAD (18). The scale had excellent internal reliability with the current sample (α = 0.93).

#### Demographic Questions

Demographic items were used to measure age, gender (male, female), sport type (individual sport, team sport), and sport achievement (international master sportsman, national master sportsman, grade 1, grade 2, grade 3, no grade).

#### Sport Injury

An item “Did you experience a sports-related injury last month” was employed to measure sport injury (0 = no, 1 = yes) (4). A sport injury refers to those injuries resulting in athletes to stop, limit, or modify sport training or competitions for at least 1 day (19).

#### ADHD

The validated Chinese version of the Adult ADHD Self-Report Scale was employed to assess ADHD symptoms (20). A sample item is “How often do you have problems remembering appointments or obligations.” Participants rated the 6-item scale on a 5-point scale (0 = never, 4 = very often). A total score was calculated, with a higher total score indicates a greater risk of being diagnosed with ADHD. The internal reliability of the scale was supported in the present study (α = 0.83).

#### Fear of Failure

The validated Chinese version of the Performance Failure Appraisal Inventory—Short Form was used to assess fear of failure in sport (21). Participants used a 5-point scale (1 = do not believe at all, 5 = believe 100% of the time) to provide responses to the five scale items (e.g., “When I am failing, I worry about what others think about me”). A higher mean scale score suggests a higher level of fear of failure. The scale had good internal reliability in the current study (α = 0.81).

#### Mental Toughness

The validated Chinese version of the Mental Toughness Inventory was used to measure mental toughness (22). The scale consists of eight items (e.g., “I consistently overcome adversity”). Participants provided responses on the scale using a 7-point scale (1 = false, 100% of the time, 7 = true, 100% of the time). A higher mean scale score indicates a greater mental toughness level. The scale showed good internal reliability with the present sample (α = 0.89).

#### Satisfaction in Sport

The validated Chinese version of the Satisfaction in Training and Competition was employed to measure satisfaction in sport (23). The scale has six items (e.g., “I am satisfied with my training and competition”). Participants answered the items using a 7-point scale (1 = strongly disagree, 7 = strongly agree). Higher scale scores represent greater levels of satisfaction in sport. The scale demonstrated good internal reliability with the current sample (α = 0.84).

### Statistical Analysis

Five item-level missing values were replaced with item means. Descriptive statistics were used to describe features of the study variables. The prevalence of GAD was calculated as the number of participants with GAD symptoms divided by the total number of respondents. Zero-order correlations were computed to determine the associations between predicting factors and GAD levels (total score). Factors that showed a significant association with GAD levels were used in subsequent multiple regression analyses. Assuming a medium and practically meaningful effect (f^2^ = 0.15, α = 0.05, power = 0.95, number of predictors = 8), a minimum sample size of 160 was needed for multiple regression analysis using G^*^Power 3 (24). Univariate/multivariate logistic regression analyses were not employed as there was inadequate statistical power for conducting these analyses with the current sample size. A statistical significance level was set at *p* < 0.05. All the analyses were conducted with IBM SPSS Statistics 25 (IBM, Armonk, NY, USA).

## Results

### Participant Characteristics and Descriptive Statistics

Table 1 represents the participant characteristics. The participants had a mean age of 20.30 years (SD = 1.51). Most of the participants were male (*n* = 206, 72%) and involved in an individual sport (*n* = 206, 72%). About half of the participants were grade 2 athletes (*n* = 132, 46%). Over one-third of the participants sustained a sport injury over the last month (*n* = 98, 34%). The participants reported below average levels of ADHD and GAD as well as moderate to high levels of mental toughness, fear of failure, and satisfaction in sport (see Table 1). Nearly one quarter (22%; 95% CI: 17, 27) of the participants met the diagnostic cutoff of GAD.

**Table 1:** Respondents' characteristics.

**Variable**	**M (SD)/*n* (%)**
Age (year)	20.30 (1.51)
**Gender**	
Male	206 (72%)
Female	79 (28%)
**Sport type**	
Team	79 (28%)
Individual	206 (72%)
**Sport achievement**	
National master sportsman	44 (15%)
Grade 1	109 (38%)
Grade 2	132 (46%)
**Sport injury**	
No	187 (66%)
Yes	98 (34%)
**GAD**	
No	223 (78%)
Yes	62 (22%)
ADHD (0–24)^a^	9.62 (4.74)
Fear of failure (1–5)^a^	2.66 (0.84)
Mental toughness (1–7)^a^	5.83 (0.78)
Satisfaction in sport (1–7)^a^	4.80 (1.11)
GAD (0–21)^a^	6.21 (5.20)

*GAD, generalized anxiety disorder; ADHD, attention deficit hyperactivity disorder*.

a *Possible range*.

### Zero-Order Correlations

The results of zero-order correlations indicated statistically significant associations between GAD and five variables, including sport injury, ADHD, fear of failure, mental toughness, and satisfaction in sport (*r* = −0.24–0.54, ps < 0.05; see Table 2). The magnitudes of these associations ranged from small to large (25). However, there were insignificant and trivial associations between GAD and other predicting variables, including age, gender, sport type, and sport achievement (*r* = −0.07–0.06, *p* = 0.26–0.74).

**Table 2:** Zero-order correlations among study variables.

	**1**.	**2**.	**3**.	**4**.	**5**.	**6**.	**7**.	**8**.	**9**.
1. Age	–								
2. Gender	−0.06	–							
3. Sport type	0.08	0.26^**^	–						
4. Sport achievement	−0.10	−0.18^**^	−0.29^**^	–					
5. Sport injury	0.03	−0.04	−0.15^*^	−0.04	–				
6. ADHD	0.01	−0.09	−0.14^*^	0.04	0.11	–			
7. Fear of failure	−0.04	−0.12^*^	−0.14^*^	0.02	0.004	0.40^**^	–		
8. Mental toughness	−0.12^*^	−0.11	−0.05	0.09	0.02	−0.13^*^	−0.23^**^	–	
9. Satisfaction in sport	0.01	0.08	0.15^*^	−0.03	−0.04	−0.20^**^	−0.30^**^	0.41^**^	–
10. GAD	−0.02	0.03	−0.07	0.06	0.14^*^	0.54^**^	0.48^**^	−0.22^**^	−0.24^**^

*ADHD, attention deficit hyperactivity disorder; GAD, generalized anxiety disorder. Gender (0 = male, 1 = female). Sport type (0 = team sport, 1 = individual sport). Sport achievement (0 = national sportsman, 1 = grade 1, 2 = grade 2). Sport injury (0 = no, 1 = yes)*.

* *p < 0.05 (2-tailed)*.

** *p < 0.01 (2-tailed)*.

### Risk and Protective Factors

Table 3 presents the results of multiple regression analysis. A history of injury, a high risk of ADHD, and a high level of fear of failure were significant risk factors for GAD (β = 0.10–0.40, partial coefficient = 0.12–0.42, ps < 0.05). These effects were small to moderate (25). However, higher levels of mental toughness (β = −0.09, partial coefficient = −0.10, *p* = 0.08) and satisfaction in sport (β = −0.03, partial coefficient = −0.03, *p* = 0.64) were insignificant and trivial protective factors for GAD. Overall, all the predicting factors explained 39% of the total variance in GAD, and the effect size was deemed large (25).

**Table 3:** Risk and protective factors of generalized anxiety disorders.

**Factor**	**Correlations**				***t***	***p***
	***B***	**β**	**95% CI of β**	**Partial**		
(Constant)	0.91				0.40	0.69
Sport injury	1.05	0.10	(0.002, 0.19)	0.12	2.04	0.04
ADHD	0.44	0.40	(0.29, 0.50)	0.42	4.44	0.00
Fear of failure	1.81	0.29	(0.17, 0.41)	0.31	6.46	0.00
Mental toughness	−0.60	−0.09	(−0.21, 0.03)	−0.10	−1.73	0.08
Satisfaction in sport	−0.12	−0.03	(−0.14, 0.07)	−0.03	−1.00	0.64

*B = unstandardized coefficient. β = standardized coefficient. Partial = standardized partial coefficient. CI, confidence interval; ADHD, attention deficit hyperactivity disorder. Sport injury (0 = no, 1 = yes)*.

## Discussion

In responding to the call to study the mental health of elite athletes (1, 2), the present research examined the prevalence of GAD symptoms and its associated risk and protective factors in elite collegiate athletes. The prevalence of GAD symptoms was 22% in our sample, which is higher than the prevalence rates measured in general populations (5%), elite national athletes (7%), and elite rugby league players (15%) based on the same measure and cutoff (26–28). The observed differences may be attributed to varying participant demographics across these studies. The finding suggests that regular screening for GAD symptoms in elite collegiate athletes should be used.

Although early research showed that female and younger athletes were inclined to have a higher anxiety level than male and older athletes (2, 29), age and gender did not significantly predict GAD in the present research. The discrepancy may be explained by the small age range in the present sample consisting of only young athletes (17–25 years) and different psychosocial factors associated with gender (30). Despite females are more vulnerable to stress-related psychopathology than males, other psychosocial factors (e.g., gender equity and marriage status) that may contribute to the development of anxiety disorders were not measured here (31).

We did not find sport type and sport achievement as significant predictors of GAD. The finding provides the initial evidence about the predictability of these two factors on GAD symptoms. Early research also showed that there was no group difference in general anxiety symptoms (non-GAD) between individual and team sport athletes (32). Of note, individual sport and low sport achievement were, however, identified as risk factors of psychological distress and depressive symptoms in elite athletes (11, 16). Thus, these two risk factors may still need to be considered for mental health enhancement in elite athletes.

In line with early research, a history of sport injury was identified as a risk factor of GAD (28, 29). A large proportion of our participant sustained a sport injury over the past month (34%), and some of them seemed not to respond to the injury in a healthy way. Sport injuries could trigger problematic responses, such as alteration of appetite and sleep disturbance, which subsequently result in the increase of anxiety levels (33). Another identified risk factor in the present research was ADHD. Indeed, non-athletic groups with ADHD were found to have a high risk of developing anxiety disorders (14). Although some symptoms of ADHD, such as impulsivity, are believed to be associated with better sport performance (e.g., a shorter reaction time is associated with impulsivity), they can cause academic difficulties and other dysfunctions (34).

One significant strength of the current research was to include several psychological predictors of GAD (fear of failure, satisfaction in sport, and mental toughness). The participants with a higher level of fear of failure were more likely to have a greater level of GAD. The finding is parallel to the study finding in that fear of failure was perceived as a risk factor of mental disorders in elite athletes (12). The sport environments are competitive in nature (e.g., peer comparison) and are usually associated with threat appraisal, which can lead to negative psychological outcomes, such as anxiety and depression (15, 35).

Given careers dissatisfaction has been found to predict anxiety (2, 36, 37), it was reasonable to expect that satisfaction in sport would be a protective factor of GAD. In agreement with the hypothesis, our finding showed that satisfaction in sport had a significant bivariate association with GAD. Mental toughness was another significant bivariate correlate of GAD in the present research. Mental toughness may represent a positive indicator of mental health (38), and early research showed that higher levels of mental toughness were associated with lower levels of psychological distress and depression (17). These findings add to the existing literature in that mental toughness could be a protective factor for several mental disorders in athletes. However, it is worthy to note that satisfaction in sport and mental toughness were no longer significant predictors of GAD after controlling for other factors. Meanwhile, the other three risk factors including sport injury, ADHD, and fear of failure remained as significant predictors of GAD. Thus, priority may be given to monitor and intervene these three risk factors.

### Limitations and Future Directions

A few limitations inherent within the present study should be acknowledged. Firstly, despite the high response rate, the participants of the present study were elite collegiate athletes from four public universities in China. Consequently, the generalizability of our study findings is limited. Sample representative of sub-elite athletes and athletes in other contexts and countries should be investigated to see whether the present findings can be replicated in future research. Secondly, as a cross-sectional survey design was employed in this study, the causal relationships between the study variables should be inferred with caution. It is suggested that future research should use a prospective longitudinal or trial design to further examine their relationships. A large sample size would be needed if the diagnosis of GAD is used as the dependent variable (i.e., to ensure adequate statistical power for conducting logistic regression analyses). Thirdly, self-reported measures were exclusively employed in the present study so that participants' responses may be biased (e.g., recalling difficulty and social desirability). Thus, objective measures or observational tools may be used in the future. Furthermore, in addition to using the GAD-7 as a screening tool, clinical diagnosis should be followed to determine the presence/absence of GAD symptoms. Finally, although several predictors of GAD were initially identified in the present research, future research may examine other potential predicting factors, such as social context factors and personality traits (3).

### Conclusions

The present research examined the predictors of GAD in a sample of elite collegiate athletes. About 2 in 10 athletes showed signs of GAD, suggesting the necessity to understand the GAD symptoms in elite collegiate athletes. A history of injury, a high risk of having ADHD symptoms, and a high level of fear of failure were risk factors of GAD. While these identified risk factors of GAD might be used for improving GAD symptoms of elite collegiate athletes, further research is needed to better understand and support the mental health for this target group.

## Data Availability Statement

The raw data supporting the conclusions of this article will be made available by the authors, without undue reservation.

## Ethics Statement

The studies involving human participants were reviewed and approved by Human Research Ethics Committee of China University of Geosciences (Wuhan). Written informed consent to participate in this study was provided by the participants, and if necessary their legal guardian/next of kin.

## Author Contributions

CL, RF, and GL conceived the overall study design. CL and GL analyzed the data. All the authors were involved in the manuscript preparation and approved the final version for submission.

## Conflict of Interest

The authors declare that the research was conducted in the absence of any commercial or financial relationships that could be construed as a potential conflict of interest.
